# Serotonin Syndrome versus Cannabis Toxicity in the Emergency Department

**DOI:** 10.5811/cpcem.2020.1.45410

**Published:** 2020-03-02

**Authors:** Jacob W. Baltz, Lamanh T. Le

**Affiliations:** * CoxHealth System, Department of Emergency Medicine, Springfield, Missouri; † CoxHealth System, Department of Pharmacy, Springfield, Missouri

## Abstract

As more states legalize marijuana, the potential of marijuana abuse could lead to an increase in the number of emergency department (ED) visits. We describe two patients who presented to the ED with dilated pupils, rigidity in both lower extremities, and clonus in both feet after inhaling the vapor of a highly potent form of marijuana. Serotonin syndrome diagnosis was initially considered in the differential diagnosis. Ultimately, high-potency marijuana abuse was the final diagnosis. Therefore, marijuana toxicity should be considered in ED patients who present with signs and symptoms similar to that of serotonin syndrome.

## INTRODUCTION

As the legalization of cannabis becomes prevalent in the United States, effects from its abuse will result in an increase in emergency department (ED) visits.^1^ We have witnessed a growing trend in our community ED among adolescents abusing a highly potent form of marijuana, butane hash oil (BHO). BHO is a concentrated form of tetrahydrocannabinol (THC) that is created by using liquid butane as a solvent to extract THC from marijuana plants. As butane is highly flammable, reports of burns and explosions have been reported from the synthesis and use of BHO. A popular trend called “dabbing” involves heating the concentrated oil and inhaling the resultant vapors. These vapors contain very high concentrations of THC, as high as 90% pure. Adolescents may use e-cigarette devices to abuse BHO as a delivery device. Such devices are easily concealed and produce almost no odor, thus leading to the potential for abuse at school and in the home.^2^,^3^

Previous case reports have shown BHO abuse may lead to agitation along with neurotoxicity and cardiotoxicity.^3^,^4^ Since THC may activate serotonin receptors and inhibit serotonin reuptake, its abuse in high concentrations may mimic serotonin syndrome.^5^ We present two cases of adolescents with recent “dabbing” use who exhibited signs and symptoms of serotonin syndrome.

## CASE REPORT

### Case 1

A 17-year-old female presented to a large community ED by emergency medical services (EMS) from her home for a possible seizure. EMS providers had witnessed agitation, altered mental status, tachycardia, muscle stiffness and tremors in the limbs, and administered 10 milligrams (mg) of midazolam intranasally. History was obtained from the EMS providers and the patient’s parents who were present in the room. The patient had been taking sertraline 50 mg daily and had also been prescribed a short course of cyclobenzaprine 5 mg every eight hours, as needed, for “muscle aches.” According to the parents, the patient had taken “a few” but stopped the cyclobenzaprine as it was not effective. No history of drug overdose or recent illness was obtained.

Upon arrival to the ED, the patient was obtunded (likely secondary to benzodiazepine), but would occasionally follow commands. Her Glasgow Coma Score was eight, scoring two points for eye-opening response, two points for verbal response, and four points for motor response. Vital signs revealed blood pressure of 135/81 millimeters of mercury (mmHg), pulse 124 beats per minute (bpm), rectal temperature of 99.6 degrees Fahrenheit (F), and 97% pulse oximetry on room air. Physical exam revealed dilated pupils of six millimeters (mm), normal neck exam, normal lung sounds, a soft and non-tender abdomen, and normal heart sounds. A neurological exam revealed rigidity in both lower extremities with a sparing of rigidity in the arms. Deep tendon reflexes showed sustained clonus in both feet, and the presence of hyper-reflexivity in the patella tendons bilaterally but with normal reflexes in the upper extremities.

Lab results showed a normal complete blood count, normal creatine kinase, normal comprehensive metabolic profile, normal arterial blood gas, normal prolactin level, and a urine drug screen positive for THC. Electrocardiogram showed sinus tachycardia, and a non-contrasted head computed tomography was normal. Serotonin syndrome was considered in the differential diagnosis. After pediatric critical care and pediatric neurology consultation, one oral dose of cyprohepatidine 4 mg was administered. The patient was admitted to the pediatric intensive care unit. Magnetic resonance imaging of the brain was normal, and an electroencephalogram showed no epileptic activity. The patient rapidly improved and was discharged the following day. Prior to discharge, the patient admitted to “dabbing” about 30 minutes prior to arrival to the hospital. The same patient returned to the ED the following night with a similar presentation, once again associated with dabbing.

### Case 2

A 16-year-old male took “a hit from a dab pen” while on the bus to school. He developed altered mental status and was transported to the ED. On arrival he was mildly obtunded, Glasgow Coma Score was 13 (three for eye-opening response, four verbal response, and six motor response). Vital signs were recorded as blood pressure 152/86 mmHg, pulse 116 bpm, oral temperature 98.6° F and 100% pulse oximetry on room air. Physical exam showed dilated pupils to five mm, tachycardia, and rigidity of the lower extremities with non-sustained clonus in the legs bilaterally. Lab results were normal with the exception of a drug screen positive for THC. This patient slowly improved over six hours of observation in the ED and was discharged home.

## DISCUSSION

Psychotic states, cardiac toxicity, and neurotoxicity have been reported as clinical sequelae of THC-induced toxicity.^4^,^6^ Our cases show additional harmful side effects of highly concentrated THC when abused by adolescents in its vapor, or “dabbing” form. Although the cases did not show all of the hallmarks of a true serotonin syndrome, some overlap existed in physical exam findings. Serotonin syndrome may show vital sign abnormalities such as tachycardia, hypertension, and hyperthermia. Physical exam findings of serotonin syndrome may reveal agitation, ocular clonus, dilated pupils, tremor, deep tendon hyper-reflexia, muscle clonus, dry mucus membranes, and flushed skin with diaphoresis.^7^

The most striking exam finding in these two ED patients was the lower extremity rigidity with hyper-reflexivity. Animal studies have demonstrated that potent cannabinoid receptor agonists may activate the serotonin receptors (5-hydroxytryptamine_1A_ and 5-hydroxytryptamine_2A_), and THC inhibits serotonin re-uptake.^5^, ^8^ Therefore, it is likely that emergency physicians may see some of the hallmarks of serotonin syndrome in “dabbing” users.

**CPC-EM Capsule**

What do we already know about this clinical entity?

Serotonin syndrome and marijuana abuse are recognizable conditions encountered in the practice of emergency medicine.

What makes this presentation of disease reportable?

We report two cases of high-potency marijuana abuse that mimicked serotonin syndrome.

What is the major learning point?

When encountering potential serotonin syndrome, a thorough social history and drug testing may be needed to rule out a disease mimic.

How might this improve emergency medicine practice?

As legalized marijuana becomes more prevalent, emergency physicians should be aware of this disease mimic.

## CONCLUSION

Medical marijuana and cannabidiol have been used and proved to be medically safe and effective; however, as marijuana use grows there is increased access of cannabinol products, including high-concentrate THCs. Our cases reflect that adolescents who abuse THC by heating and then inhaling the concentrated vapor, can present with signs and symptoms that mimic serotonin syndrome. For that reason, high-potency marijuana abuse should be considered when encountering young adults in the ED with these exam findings.
